# Research progress on delayed flowering under short-day condition in *Arabidopsis thaliana*

**DOI:** 10.3389/fpls.2025.1523788

**Published:** 2025-03-07

**Authors:** Yunhui Wang, Tianxiao Lv, Tian Fan, Yuping Zhou, Chang-en Tian

**Affiliations:** Guangdong Provincial Key Laboratory of Plant Adaptation and Molecular Design, School of Life Sciences, Guangzhou University, Guangzhou Higher Education Mega Center, Guangzhou, China

**Keywords:** flowering, short-day, CONSTANS, flowering locus T, gibberellin

## Abstract

Flowering represents a pivotal phase in the reproductive and survival processes of plants, with the photoperiod serving as a pivotal regulator of plant-flowering timing. An investigation of the mechanism of flowering inhibition in the model plant *Arabidopsis thaliana* under short-day (SD) conditions will facilitate a comprehensive approach to crop breeding for flowering time, reducing or removing flowering inhibition, for example, can extend the range of adaptation of soybean to high-latitude environments. In *A. thaliana*, CONSTANS (CO) is the most important component for promoting flowering under long-day (LD) conditions. However, CO inhibited flowering under the SD conditions. Furthermore, the current studies revealed that *A. thaliana* delayed flowering through multiple pathways that inhibit the transcription and sensitivity of FLOWERING LOCUS T (FT) and suppresses the response to, or synthesis of, gibberellins (GA) at different times, for potential crop breeding resources that can be explored in both aspects. However, the underlying mechanism remains poorly understood. In this review, we summarized the current understanding of delayed flowering under SD conditions and discussed future directions for related topics.

## 1 Introduction

Plant flowering is regulated by several environmental and endogenous conditions, which directly influence crop yield and quality. Most of the current research focuses on long-day (LD) not short-day (SD) to regulate flowering and it is particularly important to improve the mechanism of SD flowering network in some important crops. For example, the discovery and application of the long juvenile period has improved the status quo of soybean’s extremely low yields at low latitudes, which has led to the expansion of soybean to low latitudes and large-scale cultivation, and the inhibition of flowering has played a decisive role. The problem is that when only different latitude regions are planted to grow crops, especially for photoperiod-sensitive crops, timely flowering germplasm resource is needed (Lai et al., 2023), so mining flowering factors and realizing the precise flowering of crops through modern scientific means is a very important task. *A. thaliana* is a facultative long-day (LD) plant that exhibits a photoperiodic response that promotes flowering in LD (longer than 12 h of light) and delays flowering in SD (8-h light/16-h dark or 10-h light/14-h dark) (Takagi et al., 2023). *A. thaliana* regulates flowering by integrating information from multiple pathways, including photoperiodic, temperature, gibberellin (GA), vernalization, age and autonomous pathways (Fornara et al., 2010). Information from multiple pathways is integrated to regulate floral integrators that promote flowering. The photoperiod, or day length, is a stable external condition that plants can perceive and play a crucial role in controlling flowering time (Kinmonth-Schultz et al., 2023). As a florigen in plants, the FLOWERING LOCUS T (FT) protein functions as a systemic signal that induces flowering in the shoot apex (Zhu et al., 2021). The study of the key molecular mechanisms underlying the promotion of flowering in LD compared with the inhibition of flowering in short days (SD) is more in-depth in *A. thaliana*. The promotion of flowering by LD is primarily achieved through regulation of *FT* expression. The B-box (BBX) transcription factor CONSTANS (CO) is regarded as the most pivotal regulator of gene expression in *A. thaliana* (Imaizumi, 2010). The CO protein exhibited stabilization in the afternoon under LD conditions, yet only slight accumulation around ZT8 under SD conditions (Fernandez et al., 2016; Hayama et al., 2017; Valverde et al., 2004). The discrepancy in CO protein stability can account for the discrepancy in *FT* transcript levels between the LD and SD conditions (Fernandez et al., 2016). Notably, CO, which functions as a flowering promoter under LD conditions, delays flowering under SD conditions (Luccioni et al., 2019). Genetic evidence indicates that the TERMINAL FLOWER 1(TFL1) mutant *tfl1* is epistatic to *co* and *ft* is epistatic to *tfl1*. CO may increase the response to FT by shifting *TFL1* expression out of the peak of the maximal sensitivity to FT (Luccioni et al., 2019).

The delayed flowering observed under SD conditions necessitates the collective involvement of multiple repressors that exert pronounced inhibitory effects on flowering. The transcription factor SHORT VEGETATIVE PHASE (SVP) plays a pivotal role in the repression of GA biosynthesis and the expression of flowering integration factor genes FT, SUPPRESSOR OF OVEREXPRESSION OF CONSTANS 1(SOC1), and others (Andrés et al., 2014). *A. thaliana* plants can flower under SD conditions. The GA pathway is believed to play a crucial role in promoting flowering in *A. thaliana* (Wilson et al., 1992). However, the molecular mechanism by which SD delays flowering in *A. thaliana* is not yet fully understood, particularly when compared to the well-studied mechanism by which LD promotes flowering. This study presents a review of the literature on the inhibition of flowering in *A. thaliana* under SD to elucidate the underlying molecular mechanisms. It also serves as a reference for further research and breeding in the field of plant-flowering regulation.

## 2 CO-dependent regulations

### 2.1 *co* mutants and phenotypes

CO proteins are so important for the function of flowering, but there is still controversy about the phenotypes in *CO* mutants in *A. thaliana* in SD, so it is necessary to make a detailed list of the current *CO* mutants and phenotypes here, which will help us to discuss the role of CO under SD condition.

Twelve CO mutants exhibiting diverse backgrounds and mutagenesis methods were identified (**Table 1**). Most of them displayed an early flowering phenotype under SD conditions.

**Table 1:** Covered in the paper mutant phenotypes and their flowering responses under SD versus LD conditions.

Gene	mutant	Flowering phenotype		References
		SD	LD	
CO-dependent Regulations				
***CO***	*co-1(Ler)*	Early	Late	Redei, 1962
	*co-2(Ler)*	Early	Late	Luccioni et al, 2019
	* *	No phenotype	Late	Zhou and Ni, 2009
	*co-9*(Col)	Early	Late	Luccioni et al, 2019
	* *	No phenotype	Late	Johansson and Staiger, 2014
	*co-10(*Col)	Early	Late	Fernandez et al., 2016
	* *	No phenotype	Late	Jang et al., 2008
	*co-11*(Col)	Early	Late	Ning et al., 2019
	*co-12*(Col)	Early	Late	Yu et al., 2024
	*co-101*(Col)	No phenotype	Late	Arongaus et al., 2018
Circadian Clock Components				
***LHY***	*lhy-12*	Early	Early	Mizoguchi et al., 2002
*** ***	*lhy-7*	Early	Early	Park et al., 2016
***CCA1-1***	*cca1-1*	No phenotype	No phenotype	Niwa et al., 2007
*** ***	*lhy-12/cca1-1*	Early	Late	Mizoguchi et al., 2002
***PRR7***	*prr7-11*	Early	Late	Nakamichi et al., 2007
***PRR5***	*prr5-11*	Late	Late	Ito et al., 2008
***PRR7&5***	*prr7/prr5*	Early	Late	Nakamichi et al., 2007
***PRR9&7***	*prr9/prr7*	Late	Late	Nakamichi et al., 2007
***PRR9&7&5***	*prr9/prr7/prr5*	Late	Late	Nakamichi et al., 2007
***ELF3***	*elf3*	Early	Early	Zagotta et al., 1996
*** ***	*elf3-8*	Early	Early	Yu et al., 2008
***ELF4***	*elf4*	Early	Early	Lin et al., 2019
***TOC1***	*toc1-2*	Early	No phenotype	Ito et al., 2008
***LWD1&LWD2***	*lwd1/lwd2*	Early	Early	Wu et al., 2008
***FKF1***	*fkf1,fkf1-2*	No phenotype	Late	Song et al., 2012
***GI***	*gi-2*	Late	Late	Sawa et al., 2007
***CDF1&2&3&5***	*cdf1/2/3/5*	Early	Early	Fornara et al., 2009
***FBH1&2&3&4***	*fbh1/2/3/4*	Late	Late	Ito et al., 2012
***JMJ28***	*jmj28*	Late	Late	Hung et al., 2021
Light Signaling Related Regulators				
***phyB***	*phyb*	Early	Early	Lazaro et al., 2015
***phyB&CO***	*co-2/hy1*	Early	Late	Putterill et al., 1993
***HOS1***	*hos1-2*	Early	Early	Lazaro et al., 2012
***ZTL***	*ztl-105*	Early	No phenotype	Takase et al., 2011
***CRY2***	*cry2*	Early	Late	Endo et al., 2007
***CRY1***	*cry1-L407F*(gain of function)	Early	Early	Exner et al., 2010
***phyA***	*phyA*	Late	Late	Johnson et al., 1994
*** ***	*phya-211*	No date	No phenotype	Song et al., 2018
*** ***	*phya-201*	No date	Late	Song et al., 2018
***RUP2***	*rup2*	Early (+UVB)	No date	Arongaus et al., 2018
***COP1***	*cop1*	Early	No date	Jang et al., 2008
***SPA1***	*spa1-7*	Early	No phenotype	Laubinger et al., 2006
***CUL4***	*cul4cs*	Early	No phenotype	Chen et al., 2010
***TOEs***	*toe1*	Early	Early	Zhang et al., 2015
***SK12***	*sk12*	Early	Early	Chen et al., 2020
FT-Dependent Regulations				
***FT***	*ft-1*	No phenotype	Late	Balasubramanian et al., 2006
	*ft-2*	No phenotype	Late	Balasubramanian et al., 2006
	*ft-3*	No phenotype	Late	Balasubramanian et al., 2006
	*ft-10*	No phenotype	Late	Balasubramanian et al., 2006
Epigenetic Modification Related Factors of FT				
***LHP1***	*lhp1-3*	Early	Early	Chen et al., 2020
***IMPα-1,2,3***	*impα triple*	Early	Early	Chen et al., 2020
***LIF2***	*lif2*	Early	Early	Latrasse et al., 2011
FLC-FT				
***FLC***	*flc-3*	Early	No phenotype	Zhou and Ni, 2009
Vernalization Pathway				
***EFS***	*efs*	Early	Early	Kim et al., 2005
***MSI1***	*msi1-cs(overexpression)*	Early	No phenotype	Schönrock et al., 2006
***ELF7***	*elf7-2*	Early	Early	He et al., 2004
***VIP4***	*vip4-2*	Early	No phenotype	Zhang and Van Nocker, 2002
***VIP5***	*vip5-1*	Early	No phenotype	Oh et al., 2004
***VIP6(ELF8)***	*elf8-1*	Early	Early	He et al., 2004
Autonomous Pathways				
***FCA***	*fca*	Late	Late	Koornneef et al., 1991
***FLD***	*fld*	Late	Late	Chou and Yang, 1998
***FPA***	*fpa*	Late	Late	Koornneef et al., 1991
***FVE***	*fve*	Late	Late	Koornneef et al., 1991
***FY***	*fy*	Late	Late	Koornneef et al., 1991
***FLK***	*flk*	Late	Late	Boss et al., 2004
***LD***	*ld*	Late	Late	Lee et al., 1994
Other Regulators				
***COP10***	*cop10-4*	Early	No phenotype	Kang et al., 2015
***DET1***	*det1-1*(weak)	Early	Early	Kang et al., 2015
***SHB1***	*shb1*	Late	Late	Zhou and Ni, 2009
***SDR6***	*sdr6*	Late	Late	Xing et al., 2014
***ESD1***	*esd1*	Early	Early	Martin-Trillo et al., 2006
Temperature Pathway				
***SVP***	*svp-41*	Early	Early	Andrés et al., 2014
***JMJ13***	*jmj13*	Early(28℃)	Early	Zheng et al., 2019
***ELF6***	*elf6-1*	Early	No phenotype	Noh et al., 2004
***REF6***	*ref6-1,ref6-3*	Late	Late	Noh et al., 2004
***JMJ30&32***	*jmj30/jmj32*	Early	Early	Gan et al., 2014
Red Light With FT				
***SRR1***	*srr1-1*	Early	Early	Johansson and Staiger, 2014
***HRB1***	*hrb1*	Late	Late	Kang et al., 2007
***PEF1***	*pef1*	Early	Early	Ahmad and Cashmore, 1996
Other Members Of PEBP Family				
***TSF***	*tsf*	Late	Late	Yamaguchi et al., 2005
***TFL***	*tfl*	Early	Early	Yamaguchi et al., 2005
The MADS-box Family				
***MAF1***	*35S::MAF1*	Late	No phenotype	Ratcliffe et al., 2001
*** ***	*flm-3*	Early	Early	Pose et al., 2013
***VIL1***	*vil1*	Late	No phenotype	Sung et al., 2006
***HDA5***	*hda5-1*	Late	Late	Luo et al., 2015
***HDA6***	*axe1*	Late	Late	Luo et al., 2015
***VIL2***	*vil2*	Late	No phenotype	Kim and Sung, 2010
***AtRING1A***	*atring1a*	Late	Late	Shen et al., 2014
***AGL6***	*agl6-D(35s)*	Early	Early	Yoo et al., 2010
***AGL19***	*agl19*	Late	No phenotype	Schönrock et al., 2006
***AGL20(SOC1)***	*agl20-1*	Late	Late	Borner et al., 2008
***AGL24***	*agl24-1*	Late	Late	Liu et al., 2008
BBX Family				
***BBX4(COL3)***	*col3*	Early	Early	Datta et al., 2006
***BBX5(COL4)***	*col4*	Early	Early	Steinbach, 2019
***BBX24***	*sto-1*	Late	Late	Liu et al., 2014
***BBX32***	*BBX32-AMI #3*	Late	Late	Tripathi et al., 2016
*** ***	*BBX32-OX*	Late	Late	Tripathi et al., 2016
Gibberellin-dependent Regulation				
***GA1***	*ga1-3*	No flower	No flower	Reeves and Coupland, 2001
***SPY***	*spy-1*	Early	Early	Jacobsen and Olszewski, 1993
***DELLAs***	*dellaP*	Early	Early	Park et al., 2013
***BOI***	*boiQ*	Early	Early	Park et al., 2013
***NFL***	*nfl*	No flower	No phenotype	Sharma et al., 2016
***HDC1***	*hdc1*	Early	Late	Ning et al., 2019
***HDA19***	*hda19*	Early	Late	Ning et al., 2019

The *co-1* mutant was created using X-ray mutagenesis in 1962, and has been documented to flower prematurely in SD (Redei, 1962). The *co-12* mutant site was identical to that of *co-1*, except for the genetic background of the mutant. *co-12* is a Columbia (Col) background mutant, whereas *co-1* is a Landsberg *erecta* (L*er*) background mutant. Both mutants exhibit early flowering in SD (Redei, 1962; Balasubramanian et al., 2006; Zhang et al., 2015; Yu et al., 2024).

While *co-2*, which has been mutated at the carboxyl-terminus of the first B-BOX domain, has been observed to convert arginine to histidine in a L*er* background (Robson et al., 2001), *co-2* has been found to flower earlier than the wild-type (WT) plant under an 8-hour photoperiod and slightly earlier under a 10-hour photoperiod (An et al., 2004; Balasubramanian et al., 2006; Datta et al., 2006; Luccioni et al., 2019).

*co-9*, *co-10*, and *co-11* are T-DNA insertion mutants isolated from a Col background that flowered early in SD (Balasubramanian et al., 2006; Luccioni et al., 2019; Ning et al, 2019). However, further investigation revealed that *co-10* exhibited flowering patterns that differed from those of the wild type at both 21°C and 27°C in SD, suggesting that temperature may play a role in CO-mediated flowering (Fernandez et al., 2016).

The *co-1, co-2, co-9, co-10, co-11*, and *co-12* plants displayed varying degrees of early flowering phenotypes in SD. The flowering phenotype of the *co* mutants in SD may also be related to the ecotype, with the Col background exhibiting a more pronounced early flowering phenotype and the L*er* background displaying a milder early flowering phenotype. Notably, *co-2* (Martin-Trillo et al., 2006; Zhou and Ni, 2009), *co-9* (Johansson and Staiger, 2014), *co-10* (Jang et al., 2008; Laubinger et al., 2006), and *co-101* (Arongaus et al., 2018) have been shown to lack early flowering phenotypes in SD, which may be attributed to disparate culture conditions. The early flowering phenotype of the *co* mutants under SD conditions depended on *TFL1* and *FT*. However, the function of CO under SD conditions is not achieved through a reduction in *FT* transcript levels (Luccioni et al., 2019). Further investigations are required to elucidate the mechanism of CO function under SD conditions. Further understanding of the role of CO in flowering regulation under SD conditions may be achieved by investigating the relevant regulators that alter CO transcript levels and protein levels/stability.

Therefore, CO has the function of inhibiting flowering under SD, although not strongly. Based on the fact that CO has the function of integrating information from circadian clock and and light signaling, it is therefore important for us to be able to use CO as a cue to mine the factors regulating CO under SD that have the potential to refine the network of SD inhibition of flowering. The transcriptional level of *CO* is primarily regulated by the circadian clock, whereas its protein level and stability are controlled by light signals (Suarez-Lopez et al., 2001; Valverde et al., 2004). Although the key components of the circadian clock have been demonstrated to regulate CO proteins during LD (Hayama et al., 2017), there is a paucity of evidence regarding the detection of CO protein levels in mutants of the key components of the circadian clock during SD.

### 2.2 CO transcription regulators: circadian clock components

**Figure 1:**
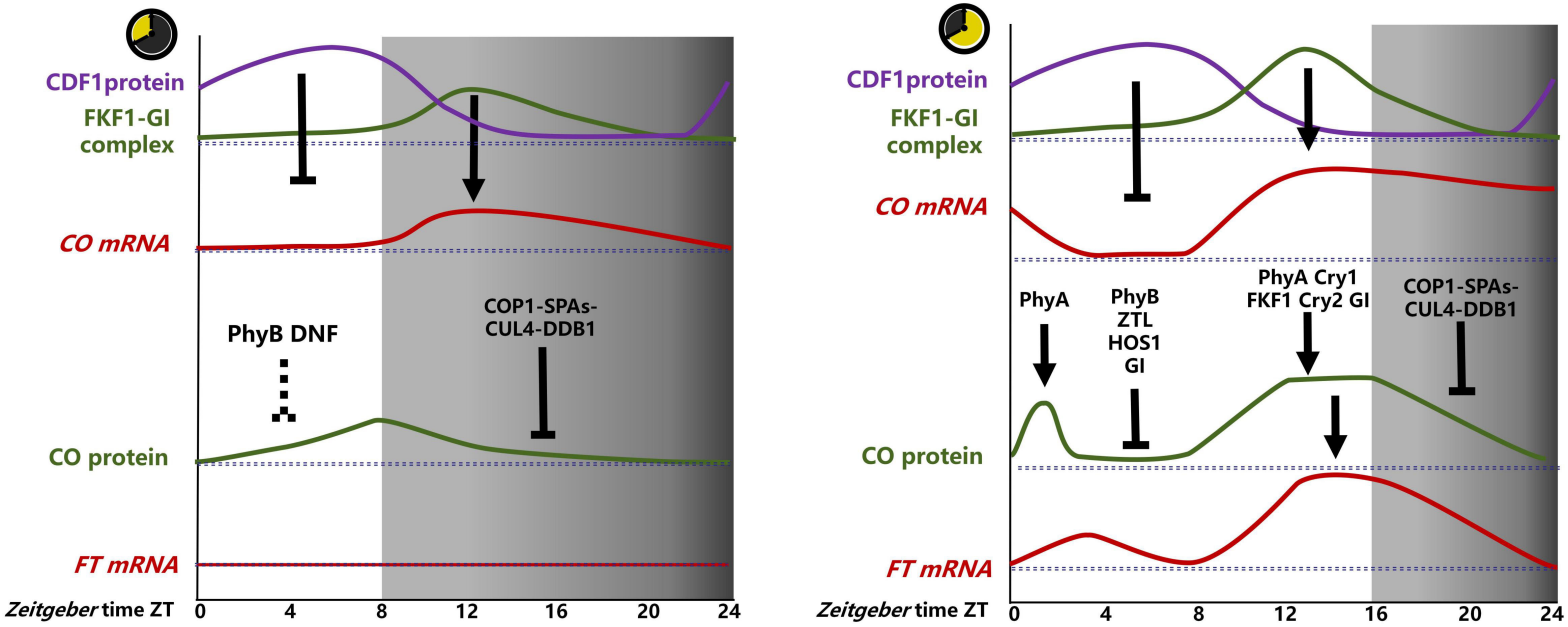
Photoperiodic regulation of CO-FT in *A. thaliana. The CO* transcript levels are mainly repressed by CDF1, which is mainly repressed by the FKF1-GI complex. The FKF1-GI complex accumulated more in ZT12-16 in LD and less in ZT12-16 in SD, resulting in high levels of *CO* at ZT12-ZT16 in LD and low levels at ZT12-ZT16 in SD. CO proteins were subjected to complex regulation in LD, stabilized by PHYA at ZT0-ZT4, degraded by GI, PHYB, HOS1, ZTL at ZT4-ZT8, PHYA, CRY1, FKF1, CRY2, and GI at ZT8-ZT16, stabilized, degraded by COP1-SPAs in dark ZT16-ZT24, and *FT* transcription was induced by CO mainly at ZT12-ZT16. In contrast, the regulation of CO proteins is less well studied in SD and is likely degraded by PHYB and DNF at ZT0-ZT4, degraded by COP1-SPAs-CUL4-DDB1 in the dark at ZT8-ZT24, and *FT* transcription is not induced by small amounts of accumulated CO proteins at ZT4-ZT12. (→, promote; 
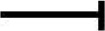
, inhabit).

The *CO* transcript remains at a low level under light (ZT0-ZT8), begins to increase after entering darkness, reaches its peak after 4 h of darkness (ZT12), and then decreases (**Figure 1**). The photoperiodic control of flowering time is inextricably linked to the circadian clock, which serves as the timing mechanism for measuring the duration of the day and night. In *A. thaliana*, complex transcriptional repression mechanisms interlocked with core clock components comprise the circadian clock (Shim et al., 2017).

Circadian clock components exhibit distinct temporal expression patterns. For instance, the primary morning-time circadian clock components include two MYB transcription factors, CIRCADIAN CLOCK ASSOCIATED1 (CCA1) and LATE ELONGATED HYPOCOTYL (LHY) (Nagel et al., 2015; Kamioka et al., 2016), as well as PSEUDO RESPONSE REGULATOR 9 (PRR9), PRR7, and PRR5 identified noon-time components (Nakamichi et al., 2010; Nakamichi et al., 2012; Liu et al., 2014; Liu et al., 2016), LUX, ELF3, ELF4, and TOC1/PRR1 as key evening- and night- time components of the circadian clock (Doyle et al., 2002; Helfer et al., 2011; Nusinow et al., 2011; Herrero et al., 2012; Huang et al., 2016). Mutants in most genes exhibit aberrant flowering times under both LD and SD conditions.

The LHY mutant *lhy-12* and CCA1 mutant *cca1-1* flowered prematurely in SD, and the double mutant *lhy-12/cca1-1* flowered especially early. This indicates that these two genes can function redundantly to inhibit flowering in SD (Mizoguchi et al., 2002)Subsequently, the expression peaks of the FLAVIN-BINDING, KELCH REPEAT, F-BOX PROTEIN 1 (FKF1), and GIGANTEA (GI) clock output, which are related to photoperiodic flowering, were advanced by approximately four hours in the *lhy-7* mutant. Conversely, when the endogenous circadian cycle of the *lhy-7* mutant aligns with the external light/dark cycle, the upregulation of *FT* is no longer observed (Park et al., 2016). This finding suggests that the early flowering observed in SD in the *lhy-7* mutant is due to aberrant expression of photoperiodic flowering genes (Park et al., 2016). The flowering phenotype of *lhy-7/co-101* under SD conditions has yet to be documented.

The midday circadian clock components are PPR9, PPR7, and PPR5. Among these, the PPR7 mutant Δ*7* flowered prematurely under SD conditions (Nakamichi et al., 2007), the PPR5 mutant *prr5-11* flowered slightly later (Ito et al., 2008), and the *prr7*/*prr5* double mutant Δ*7* flowered at the same time as Δ*7* and earlier than the PPR9/PPR/PPR5 triple mutant Δ*7* (Nakamichi et al., 2007). This indicates that PPR7 may play a dominant role in inhibiting flowering in SD. Nevertheless, no observable phenotypes have been documented for these PPR9 single mutants under SD conditions.

The evening circadian clock component ELF3 mutants, *elf3* and *elf3-8*, flowered early during SD (Yu et al., 2008; Zagotta et al., 1996). The evening circadian clock component ELF4 mutant, *elf4*, also flowered early in SD (Lin et al., 2019). Subsequent studies demonstrated that ELF4 negatively regulates *CO* expression by forming an ELF4-GI complex with GI, which segregates GI from the CO promoter to specific nucleosomes (Kim et al., 2013). Furthermore, the early flowering phenotype of *elf4* under SD conditions can be fully compensated by the overexpression of *EFL1*, partially compensated by the overexpression of *EFL3*, and not compensated by the overexpression of *EFL2* (Lin et al., 2019), indicating that *EFL1* is the primary flowering regulator through the circadian clock pathway under SD conditions.

The evening circadian clock component TIMING OF CAB2 EXPRESSION 1 (TOC1)/PRR1 mutants, *toc1* and *toc1-2*, flowered at an early stage under SD conditions (Niwa et al., 2007). LIGHT-REGULATED WD1 (LWD1) and LWD2 affect flowering onset by regulating the circadian clock. *lwd1/lwd2* double mutants exhibit an early flowering response in SD (Wu et al., 2008).

In SD, *CO* expression commences in the dark due to the accumulation of GI protein during the light period, reaching a peak at the end of the light phase. In contrast, the FKF1 protein does not reach a peak until ZT12 in the dark, resulting in the level of the GI-FKF complex remaining at a low level. This results in the accumulation of CYCLING DOF FACTORs (CDFs) proteins in light, which subsequently inhibits *CO* transcription. Following the transition to the dark, CDFs undergo degradation, allowing FBHs to bind to the E-box element in the *CO* promoter, activating *CO* transcription (Song et al., 2015; **Figure 1**).

Flowering regulation by these factors under SD conditions differed from that under LD conditions. The *fkf1* and *fkf1-2* mutants exhibited an unremarkable flowering phenotype (Song et al., 2012), indicating that FKF1 does not directly regulate flowering but that the transcript levels of *CO* and *FT* were moderately reduced in the afternoon (Imaizumi et al., 2003). The GI mutant *gi-2* exhibits a late-flowering phenotype (Sawa et al., 2007; Sawa and Kay, 2011). However, the precise mechanism by which GI promotes flowering remains unclear. However, it has been demonstrated that GI does not regulate *CO* (Sawa and Kay, 2011). In addition, FKF1-GI complexes have been observed to form in SD (Sawa et al., 2007). The overexpression line *35S∷HA-FKF1#18 35S∷GI-TAP/fkf1* exhibited an extremely early flowering phenotype (Sawa et al., 2007), This indicates that the SD of *35S∷HA-FKF1#18 35S∷GI-TAP/fkf1* could promote *CO* transcripts by enhancing the level of the FKF1-GI complex, thereby accelerating flowering (Sawa et al., 2007). Nevertheless, the *fkf1/gi* mutant does not exhibit a flowering phenotype under SD conditions (Sawa et al., 2007).

The CDF family comprised five members. The quadruple mutant *cdf1/2/3/5* exhibited an extremely early flowering phenotype in SD (Fornara et al., 2009). The FBH family comprises four members. The single mutants did not exhibit a flowering phenotype. However, *fbh* quadruple mutants displayed a slight delay in flowering compared to the wild type (Ito et al., 2012). In contrast, both *35S:FBH1* and *35S:FBH2* plants exhibit an extremely early flowering phenotype (Ito et al., 2012). Demethylase JMJ28 interacts with FBH transcription factors to activate the *CO* promoter by removing the repressor marker H3K9me2. The *JMJ28* mutant *jmj28* exhibited a slight delay in flowering under SD conditions (Hung et al., 2021).

In summary, under SD conditions, except for *prr5-11* and *gi-2*, mutants of the circadian clock component and related regulators exhibited early flowering phenotypes, particularly *toc1-2*, *elf3*, and *lhy-12/cca1-1*. This suggests that most circadian clock components are repressed in SD, Some studies have indicated that the key components of the circadian clock during LD regulate *CO* transcription and directly regulate CO protein stability (Hayama et al., 2017), The mechanism by which these genes regulate flowering under SD needs to be deepened, and the components related to the circadian clock pathway are a very good candidate for breeding targets.Furthermore, these mutants exhibited elevated levels of *FT* transcripts, suggesting that repression was achieved by the suppression *of FT* transcription.

### 2.3 CO protein regulators

CO primarily functions as a transcription factor at the protein level. The exact mechanism of how CO proteins function in SD in contrast to LD,which inhibits not promotes flowering, is not yet clear. The main regulating CO proteins are light signaling factors, so through the phenotype of the mutant and the level of CO protein in the mutant, explore the possible mechanisms by which CO proteins regulate flowering in SD.

The level of CO protein increases with prolonged illumination time, reaches its peak at the end of 8 h (ZT8), and then decreases rapidly after entering darkness (Fernandez et al., 2016; Yu et al., 2024) (**Figure 1**). In contrast the level of *CO* transcript, these findings suggest that the regulation of CO protein levels may primarily depend on degradation rather than synthesis, and is not significantly correlated with alterations in *CO* transcript levels under SD conditions.

#### 2.3.1 Light signaling related regulators

CO protein regulators have been studied more systematically under LD conditions and are mainly regulated by light signalling pathway factors, so how these factors regulate CO proteins under short day conditions and whether changes in CO proteins contribute to flowering will be discussed here.

Under LD conditions, the influence of different light signals on the photoperiodic flowering response varied. In the morning, CO degradation relies mainly on the red-light photoreceptor Phytochrome B (PhyB), PHYA and ZEITLUPE (ZTL) (Putterill et al., 1995; Reed et al., 1994; Hwang et al., 2019). In the afternoon, FKF1,Cryptochrome 1 (Cry2),Cry1,all are blue light photoreceptor, stabilize CO (Takase et al., 2011; Endo et al., 2007; Valverde et al., 2004). In the absence of light signaling at night or in the dark, CO is primarily degraded by CONSTITUTIVE PHOTOMORPHOGENIC 1 (COP1), SUPPRESSOR OF PHYA-105s (SPAs), CULLIN4 (CUL4) and Damaged DNA Binding Protein1 (DDB1) (Jang et al., 2008; Hwang et al., 2019); When the time comes to dawn, PHYA also stabilize CO transiently (Song et al., 2018; **Figure 1**).

PhyB, which receives red light, plays an important role in reducing the abundance of CO proteins and their activity in LD. *phyb* mutants exhibited early flowering in response to SD (Reed et al., 1994). The CO and PhyB double mutant *co-2/hy1* exhibited a flowering time that was earlier than *co-2* and later than *hy1*, indicating that CO is necessary for the early flowering phenotype of *hy1* under SD conditions (Putterill et al., 1995). In SD, CO proteins are degraded via a pathway that requires PhyB (Andrés and Coupland, 2012; **Figure 1**); however, there is a paucity of relevant biochemical experiments. HOS1 and PhyB have been shown to play redundant roles in the regulation of flowering during SD (Lazaro et al., 2012; Lazaro et al., 2015). Under SD conditions, the *hos1-2* mutant flowered early, whereas the *hos1-2/co-2* double mutant flowered even earlier. This suggests that the early flowering of *hos1* under SD conditions was not achieved through the CO pathway (Lazaro et al., 2012).

PhyA, which receives far-red light, contributes to CO stabilization in the dawn and afternoon under LD conditions. *phyA* flowered late in SD, which promotes flowering in SD (Johnson et al., 1994).

*ztl-105* and *ztl-4* mutants of the blue light-responsive ZTL flowered early under SD conditions (Takase et al., 2011; Hwang et al., 2019). A comparable CO protein abundance was observed in *35S:3HA-CO* and *35S:3HA-CO/ztl-4* plants (Hwang et al., 2019) This mechanism appears to be distinct from that of ZTL-degrading CO activity in the morning of an LD (Song et al., 2014).

The early flowering observed in *ztl* mutants under SD conditions depends on FKF1 (Takase et al., 2011). In addition, the flowering phenotypes of the FKF1 mutants, *fkf1* and *fkf1-2*, are not evident (Song et al., 2012), indicating that FKF1 does not independently regulate flowering under SD conditions. Nevertheless, *35S:3HA-CO* showed a early flower phenotypes, but no notable discrepancy was observed in CO protein abundance between *35S:3HA-CO* and *35S:3HA-CO/fkf1-2* (Song et al., 2012), indicating that early flowering of *35S:3HA-CO* is independent of FKF1.

CRY2, which receives blue light in the afternoon on LD, degrades the COP1-SPAs complex and stabilizes CO (Endo et al., 2007). *cry2* mutants have been observed to flowered early in SD (Endo et al., 2007)

*cry1-L407F*,a gain of function allele of CRY1, showed a very early flowering. It can increase the sensitivity of phytochrome signaling cascades (Exner et al., 2010).

As darkness decreases, plants cease to receive light signals, CONSTITUTIVE PHOTOMORPHOGENIC 1 (COP1) degradation CO protein (Jang et al., 2008). The *cop1* mutant flowers prematurely under SD conditions, and substantial accumulation of CO proteins was observed in the dark (Jang et al., 2008). The *cop1 co* mutant exhibited a flowering time that was later than *cop1* and earlier than *co*, indicating that CO is necessary for the early flowering of *cop1* (Mcnellis et al., 1994; Jang et al., 2008). The SPA family comprises four members. The SPA family comprises four members: SPA1, SPA2, SPA3, and SPA4. Of these, SPA1 plays a dominant role in flowering, and SPA1 is sufficient for normal photoperiodic flowering. Furthermore, SPA1 is essential for maintaining flowering in wild-type plants under SD conditions (Laubinger et al., 2006). The *spa1* mutant exhibited early flowering under SD conditions, which may be attributed to the presence of a substantial number of *FT* transcripts (Laubinger et al., 2006). Subsequently, considerable accumulation of CO proteins was observed in *spa1-7* mutants under dark conditions (Jang et al., 2008). Consequently, in the absence of light signaling, plants require the COP1-SPAs complex to inhibit flowering by suppressing CO (Yu et al., 2008). CULLIN4 (CUL4)-Damaged DNA Binding Protein1(DDB1) may function with COP1-SPAs complexes to regulate CO protein degradation. Mutant *cul4cs* (for CUL4 co-suppression) exhibit an early flowering phenotype in SD (Chen et al., 2010). DDB1 has two isoforms: DDB1a,DDB1b.*ddb1b* mutant is embryo-lethal, whereas the knockout line *ddb1a* exhibits no obvious phenotype (Schroeder et al., 2002), indicating that DDB1 inhibits flowering via CUL4.

Ultraviolet B (UV-B) radiation is an essential component of light. The Repressor of UV-B Photomorphogenesis 2 (RUP2) has been identified as a flowering repressor under SD conditions containing UV-B. This repressor depends on the UV-B photoreceptor UVR8 (UV RESISTANCE LOCUS 8), and represses *FT* expression by inhibiting the binding of CO to the FT promoter (Arongaus et al., 2018). The *rup2* mutant exhibited an early flowering phenotype under SD conditions (+UV-B) (Arongaus et al., 2018).

#### 2.3.2 Other regulators

Besides the previously discussed CO protein regulators, other mutants of regulators that regulate flowering through CO under LD conditions have been observed to exhibit flowering phenotypes under SD conditions. The observations are presented in the following section. The TARGET OF EAT (TOE) proteins are members of the APETALA2 (AP2)-LIKE family of proteins, which includes TOE1, TOE2, TOE3, SCHLAFMÜTZE (SMZ), and SCHNARCHZAPFEN (SNZ) (Aukerman and Sakai, 2003; Chen, 2004). During the morning of an LD cycle (ZT0-ZT4), holidays inhibit CO activity by directly interacting with it (Zhang et al., 2015). In SD, *TOE1* was expressed exclusively during the light period (ZT0-ZT8), with the highest level of expression occurring at ZT4. The *toe1* mutant flowered earlier than the wild type, whereas *toe1/co* flowered later than the wild type and *toe1* mutant (Zhang et al., 2015). This suggests that CO plays a role in the early flowering of *toe1*. Flowering was likely facilitated in the background of *toe1* in SD; TOE2 was also involved in flower formation in SD, with *toe1/toe2* flowering earlier than *toe1* and later than *toe1/toe2*/*co* flowering (Zhang et al., 2015). It has been postulated that TOE1 and TOE2 may compete redundantly with CO for the *FT* promoter, functioning as inhibitors of CO binding to FT under SD conditions. Further experiments are required to substantiate this hypothesis. SHAGGY-like kinase 12 (SK12) is a member of the glycogen synthase kinase-3 family. Under SD conditions, *sk12* also flowered early because of the inability of most of its CO proteins to be phosphorylated and their subsequent degradation through ubiquitination. This results in the accumulation of CO proteins and the promotion of flowering through the FT pathway (Chen et al., 2020). DAY NEUTRAL FLOWERING (DNF) encodes a functional membrane-bound E3 ligase, suggesting that DNF targets a repressor of CO for degradation by the proteasome pathway, *dnf* mutants flowered early (Morris et al., 2010). The GI protein is indirectly involved in the regulation of CO protein stability by forming a complex with the FKF1 and ZTL proteins under conditions of LD, and the *gi-2* allele flowered late under SD (Song et al., 2014). The flowering time of *35S:3HA-CO* was earlier than *35S:3HA-CO/gi-2 #1* and later than *35S:3HA-CO/fkf1-2 gi-2 #20* in SD (Song et al., 2014), indicating that early flowering of *35S:3HA-CO* depends on GI. However, whether GI promotes flowering in nature remains to be determined using CO.

In summary, two insights can be drawn. One is that in the mutants with a large accumulation level of CO proteins, it still led to its early flowering phenotype, so a large number of CO proteins may still have promoting flowering function in SD, such as *cop1*, *spa1*, *35S:3HA-CO*, *sk12.* And the regulation of flowering by CO proteins may be a dosage effect, which may also be why *A. thaliana* needs to maintain low levels of CO proteins in SD, and this mechanism is important for the function of CO proteins in SD. The others is the most of photoreceptor mutants showed an early flowering phenotypes, such as *phyb,ztl-105,cry2* etc. suggesting that the photoreceptors mainly inhibit flowering in SD, and this inhibition is firstly due to unable to stabilize a large number of CO proteins. However, how the photoreceptors regulate to these low levels of CO proteins in SD may be the direction of the future research.

## 3 FT-dependent regulations

*A. thaliana* exhibits delayed flowering and a notable reduction in *FT* transcription under SD conditions. Besides the CO-FT pathway, *A. thaliana* represses *FT* transcription through epigenetic modifications of FT, FLC-FT pathway, and various related FT-regulated genes.

### 3.1 *FT* mutants and phenotypes

FT is a mobile protein synthesized in the companion cells of leaves and transported to the SAM through the phloem, where it promotes flowering (Maple et al., 2024). Under SD conditions, flowering is delayed in the wild type, which does not express or express low levels of *FT* transcripts (Luccioni et al., 2019). Furthermore, *the FT* mutants *ft-1, ft-2, ft-3*, and *ft-10* do not exhibit obvious flowering phenotypes (Balasubramanian et al., 2006). In addition, natural variation in *FT*-creating promoter length does not alter flowering time in SD (Liu et al., 2014), suggesting that *FT* and FT under natural conditions in SD are not directly involved in regulating flowering.

### 3.2 Epigenetic modification related factors of *FT*

Photoperiods can directly regulate *FT* through cis-regulatory changes at its gene locus. Furthermore, under SD conditions, a minimal distance between the regulatory regions is required to fully suppress FT expression (Liu et al., 2014).

LIKE HETEROCHROMATIN PROTEIN 1 (LHP1) is a transcriptional repressor of flowering-related genes. It represses *FT* expression by directly associating with FT chromatin. The *lhp1-3* mutant flowered under SD conditions have been shown to have elevated *FT* transcripts (Chen et al., 2020). Under SD conditions, LHP1 interacts with IMPα-1, 2, 3 to regulate flowering by modulating epigenetic modifications of *FT*. *Impα-1, 2, 3* triple mutants flowered early, exhibited severely impaired nuclear targeting of LHP1, and displayed a substantial elevation of *FT* transcripts under SD conditions (Chen et al., 2020). Moreover, LHP1 interacts with LHP1-Interacting Factor 2 (LIF2) in the nucleus. LIF2 belongs to the hnRNP family of proteins and is involved in RNA processing, and *lif2* flowered early under SD conditions (Latrasse et al., 2011).

### 3.3 FLC-FT dependent pathways

FLC plays a pivotal role in regulating flowering under LD conditions by engaging numerous genetic regulatory pathways associated with flowering (Michaels et al., 2003; Whittaker and Dean, 2017). In addition, FLC inhibits flowering under SD conditions and *flc-3* exhibits early flowering (Zhou and Ni, 2009). Notably, the transcript levels of *FLC* in the wild type are comparable under LD and SD conditions (Zhou and Ni, 2009).

#### 3.3.1 Vernalization pathway

Vernalization is the process by which plants undergo prolonged low-temperature treatments to promote flowering. Vernalization primarily disengages *FLC* inhibition of flowering by repressing *FLC* expression, optimizing the timing of flowering to align with the cessation of winter and onset of spring, which allows for maximal reproductive acclimation (Whittaker and Dean, 2017). The expression levels of *FLC* in the vernalization pathway are primarily regulated by upstream *FRIGIDA (FRI)* (Shindo et al., 2005; Zhang and Jiménez-Gómez, 2020). Allelic variation at the *FRI* locus in *A. thaliana* is a significant factor influencing the natural variation in flowering time (Johanson et al., 2000; Kim and Michaels, 2006). FRI can methylate *FLC* chromatin in complex with the histone methyltransferase EARLY FLOWERING IN SDS (EFS), which promotes *FLC* expression and *efs* mutant flowered early in SD (Kim et al., 2005).

In the vernalization pathway, the Polycomb Repressive Complex (PRC2) can cause *FLC* silencing through its specific component, PLANT HOMEODOMAIN (PHD) (Whittaker and Dean, 2017). PRC2 is a polycomb group (PcG) protein complex that is a cellular memory module that maintains the repression of gene transcription (Brock and Fisher, 2005). In the PcG protein MSI1 overexpression line *msi1-cs*, there was a marked reduction in *MSI1* levels. These plants also exhibit early flowering in SD (Schönrock et al., 2006).

The RNA polymerase II-associated factor 1 (PAF1) complex also exerts a negative regulatory effect on FLC and FLC-like proteins via the vernalization pathway. The PAF1 complex comprises four subunits, ELF7, ERNALIZATION INDEPENDENCE 4 (VIP4), VIP5, and VIP6/ELF8. The *elf7-3, vip4-2, vip5-1*, and *elf8-1* mutants exhibit an early flowering phenotype (He et al., 2004; Zhang and Van Nocker, 2002). Of these, *elf7-3* and *elf8-1* exhibited an early flowering phenotype (He et al., 2004). SKIP interacts with ELF7 to regulate flowering by activating *FLC* transcription and *skip* flowered early in SD (Cao et al., 2015). Therefore, the vernalization pathway also inhibited flowering in SD.

#### 3.3.2 Autonomous pathways

The effect of FRI on *FLC* expression is antagonized by a group of proteins that have been termed the autonomous pathway, because their activity appears to be largely independent of the environment (Koornneef et al., 1998). The main components of the autonomous pathways involved in FLC are *FCA*, *FLOWERING LOCUS D (FLD)*, *FPA*, *FVE*, *FY*, *FLOWERING LOCUS K* (FLK), and LD (LUMINI DEPENDENS). Loss-of-function of these genes delays flowering under any photoperiod (Chou and Yang, 2002; Koornneef et al., 1991; Lee et al., 1994).

#### 3.3.3 Other regulators

COP10 epigenetically induces *FLC* expression by interacting with *MULTICOPY SUPPRESSOR OF IRA14 (MSI4)/FVE (MSI4/FVE)* (Kang et al., 2015). *cop10-4* flowered early under SD conditions (Kang et al., 2015). COP10, DE-ETIOLATED1 (DET1), and Damaged DNA Binding Protein1 (DDB1) interacts to form the CDD complex and inhibit photomorphogenesis in the dark (Kang et al., 2015); *det1-1* weak mutants flowered early in SD (Kang et al., 2015), suggesting that the CDD complex inhibits SD flowering. There are also genes that regulate flowering by modulating autonomous pathways. SHORT HYPOCOTYL UNDER BLUE1 (SHB1) encodes a yeast SYG1-like protein that represses the *FLC* pathway under SD conditions, resulting in *FT* transcription activation (Zhou and Ni, 2009). The *shb1* mutant flowered late, whereas the functionally acquired mutant *shb1-D* flowered early (Zhou and Ni, 2009). *SDR6* encodes a short-chain dehydrogenase/reductase containing an NAD(P) domain that regulates flowering through autonomous pathways. The *SDR6* mutant*, sdr6*, flowered late (Xing et al., 2014).HIGH PLOIDY2 (HPY2) as an E3 SUMO ligase for FLC, regulates FLC function and stability at both the transcriptional and post-translational levels through its E3 SUMO ligase activity (Kwak et al., 2016). *hpy2-2* mutants flowered early than wild-type plants (Kwak et al., 2016). EARLY IN SDS 1 (ESD1) is required for the expression of *FLC* repressors at levels that inhibit flowering. The ESD1 mutant *esd1* flowered early (Martin-Trillo et al., 2006), REF6 encodes an H3K27 demethylation transferase that represses *FLC* expression by demethylating FLC, and the REF6 mutants *ref6-1* and *ref6-2* flowered late (Noh et al., 2004).

### 3.4 Temperature pathway

*A. thaliana* can overcome delayed flowering by increasing suitable temperature under SD conditions. The 28°C *FT* transcript level in WT plants under SD conditions was over 10-fold higher than that at 23°C, and diurnal oscillations were not affected (Balasubramanian et al., 2006).

The SVP mutant *svp-41* flowered early in SD (Fernandez et al., 2016) and requires only a small amount of FT to flower at 27°C. This increased sensitivity to *FT* during flowering may be due to a reduction in SVP activity at the apex at 27°C. In addition, *svp-41 ft-10 tsf -1* plants flowered simultaneously under 21°C-SD and 27°C-SD (Fernandez et al., 2016), indicating that the flowering response to temperature was not affected by the *ft-10/tsf -*1 mutation. These findings indicate that SVP, FT, and TSF are indispensable for the thermosensory induction of flowering under SD conditions. Furthermore, SVP selectively binds to *FLOWERING LOCUS M (FLM)*- and *MADS AFFECTING FLOWERING2(MAF2)*-specific transcripts and regulates flowering through temperature (Airoldi et al., 2015; Pose et al., 2013). The SVP plays a pivotal role in delaying flowering during SD. The JUMONJI (JMJ) family members JMJ13, JMJ30, and JMJ32 regulate *A. thaliana* flowers at different temperatures through epistatic regulation of *FLC* in SD.JMJ13 is an H3K27me3 demethylase that may negatively regulate temperature-driven flowering by suppressing temperature-photoperiod compensation, *jmj13* mutant flowered early at 29°C rather than at 22°C (Zheng et al., 2019). JMJ13, along with ELF6 and REF6, influences the genome-wide distribution of H3K27me3, regulates the activation of tissue-specific genes (Pajoro et al., 2017), and plays a role in the regulation of a flowering pathway that also affects flowering in SD. *elf6-1* showed an early flowering, whereas *ref6-1* showed a late flowering (Noh et al., 2004). The *jmj30/jmj32* double mutant exhibited an early flowering phenotype when cultivated under SD conditions at 29°C. JMJ30 has been demonstrated to directly binds to *FLC*, removing the inhibitory histone modification H3 lysine 27 trimethylation (H3K27me3) (Gan et al., 2014).

*A. thaliana* can release the inhibition of *FT* through the SVP-FLM and JMJ-FLC pathways, flowering earlier under the high-temperature conditions of SD.

### 3.5 Red light with FT

SENSITIVITY TO RED LIGHT REDUCED 1 (SRR1) is a protein with reduced sensitivity to red light, which was previously involved in the regulation of the circadian clock and PhyB signaling pathway in *A. thaliana* (Staiger et al., 2003). Mutant *srr1-1* flowered early in SD (Johansson and Staiger, 2014). SRR1 suppresses *FT* expression and thus inhibits flowering under SD conditions by activating the expression of FT-binding repressors CDF1, TEM1, TEM2, and FLC (Johansson and Staiger, 2014).

Hypersensitivity to Red and Blue 1 (HRB1) mutant *hrb1* flowered late, whereas HRB1 overexpressing line flowered early in SD (Kang et al., 2007), indicating that HRB1 promotes flowering in SD. The SD flowering phenotype of *hrb1/phyB-9* was the same as that of *phyB-9*, and *hrb1/cry2* showed a phenotype similar to that of *hrb1* (Kang et al., 2007), suggesting that HRB1 mediates the regulation of flowering via red, but not blue, light signaling. *hrb1/ft-2 flowered* later than *ft-2* (Kang et al., 2007), indicating that HRB1 promotes flowering in SD. The Phytochrome-signaling Early Flowering 1 (PEF1) mutant *pef1*, screened earlier in the SD early flowering mutant, showed an early flowering phenotype (Ahmad and Cashmore, 1996).

### 3.6 Other FT-dependent regulations

#### 3.6.1 Other members of the phosphatidylethanolamine-binding protein family

FT is a member of the Phosphatidylethanolamine-binding Protein (PEBP) family, which comprises six members that can be categorized into three branches: FT-like, and TERMINAL FLOWER1 (TFL1)-like, MOTHER OF FT (MFT)-like (Chardon and Damerval, 2005). Another floral integrator, the TWIN SISTER OF FT (TSF) mutant *tsf* and its overexpression, was found in late and early flowering under SD conditions (Yamaguchi et al., 2005), although it is thought to function redundantly with FT in LD to promote flowering, and its specific mechanism under SD conditions is not clear. Mutant *tfl* flowered early under SD conditions and TFL negatively regulates the transcription of FD-dependent target genes, participating in the transcriptional repression of FT-activated genes (Hanano and Goto, 2011). To our knowledge, no study has reported the involvement of MFT in the regulation of flowering by SD.

#### 3.6.2 The MADS-box family

Besides the MADS-box family members above mentioned, MADS AFFECTING FLOWERING1 (MAF1)/FLM/AGL27, MAF4, MAF5, AGL6, AGL19, AGL20, and AGL24family members play a pivotal role in the regulation of SD flowering.

The MAF1/FLM/AGL27 is responsible for the natural variation in the SD flowering time observed in certain ecotypes (Werner et al., 2005). The *35S::MAF1* construct was shown to flower approximately one month later (Ratcliffe et al., 2001). The VERNALIZATION INSENSITIVE 3-LIKE 1 (VIL1) mutant, *vil1*, flowered only late in SD, and VIL reduces the transcript levels of *MAF1/FLM* in SD (Sung et al., 2006). Furthermore, transcriptome changes induced by warm ambient temperatures in *A.thaliana* require VIL1, and its loss-of-function resulted in insensitivity to ambient temperatures (Sung et al., 2006; Kim et al., 2023).Histone deacetylation/acetylation plays a crucial role in maintaining genomic stability, regulating transcription, and influencing plant development. Histone acetylation is controlled by histone histone acetyltransferases and histone deacetylases (HDACs or HDAs) (Liu et al., 2014b).The HDACs family member, HDA5 mutant *hda5-1*, exhibited elevated *FLC* and *MAF1* transcript levels under SD conditions, resulting in a late-flowering phenotype (Luo et al., 2015). Conversely, the HDA6 mutant *axe1* displays late flowering under SD conditions and elevated *MAF4* transcript levels (Luo et al., 2015). *MAF5* repressed the VIN3-LIKE 2 (VIL2) gene to accelerate flowering under SD conditions, the *vil2* mutant exclusively exhibits a late-flowering phenotype under SD conditions, but the *maf5* mutant showed no obvious phenotype (Kim and Sung, 2010). Furthermore, the PRC1 RING-finger protein AtRING1A promotes flowering by suppressing *MAF4* and *MAF5* expression, which downregulates two floral integrators, FT and SUPPRESSOR OF OVEREXPRESSION OF CONSTANS 1 (SOC1). The *atring1a* mutant exhibited late flowering in SD (Shen et al., 2014). AGL6 has been demonstrated to negatively regulate *FLC*, *MAF4*, and *MAF5* expression and positively regulate *FT* expression at the transcriptional level, promoting flowering in *A.thaliana agl6-D* (in which AGL6 is activated by the 35S enhancer). This results in early flowering in SD (Yoo et al., 2010). AGL19 has been identified as a negative regulator of *msi1-cs*. The AGL19 mutant *agl19* flowered late in SD (Schönrock et al., 2006). SIN3-like proteins (SNLs) and their homologous protein MSI delay the flowering time of SNLs by repressing AGL19-regulated HDA9, *snl2/3/4*, and *hda9* during early flowering in SD plants (Ning et al., 2019; Kim et al., 2013). Subsequent studies have demonstrated that HDA9 inhibits premature flowering under SD conditions by modifying the local chromatin environment and suppressing hyperactivation of the *FT* upstream activator *AGL19* (Kang et al., 2015).The *AGL20/SOC1* mutant exhibited delayed flowering time, whereas the *SOC1* overexpression line *35S::AGL20-13* displayed an exceptionally early flowering time. SOC1 is a major floral integrator that integrates both developmental and environmental cues into floral genetic networks, but under SD conditions, SOC1 is only a minor target of GA signaling at the shoot meristem (Borner et al., 2008; Galvão et al., 2012). Another MADS-box gene, *agl24*, has been demonstrated to delays flowering under SD conditions by regulating SOC1 (Liu et al., 2008).

Therefore, in SD, the *FT* repressors *MAF1*, *MAF4*, and *MAF5* are associated with delayed flowering, the *FT* activators *AGL19* and *ALG20* are associated with the promotion of flowering, and HDAs proteins are also actively involved in flowering through epigenetic modifications.

#### 3.6.3 The BBX family

Besides *CO*, several members mutants of the BBX family also exhibited flowering phenotypes. Their function in regulating flowering is related to the *FT* in SD. *BBX4/COL3* and *BBX5/COL4* inhibit flowering, and upregulation of *FT* expression has been detected in both *col3* and *col4* (Datta et al., 2006; Steinbach, 2019). In contrast, an overexpression line of *COL5* caused early flowering, and upregulation of *FT* levels was also detected; however, low levels of *COL5* expression did not affect flowering in SD (Hassidim et al., 2009). BBX24/STO mutant *sto-1* flowered late in SD, and the *STO* overexpression line *STO-OE* reduces the expression level of *FLC*, and at the same time, due to competition with *FLC*, the regulated downstream genes are not affected (Liu et al., 2014). Competition is a regulated downstream gene that activates *FT* and *SOC1* expression (Liu et al., 2014). COL3 targets FT in the presence of BBX32 to regulate the flowering pathway, but both *BBX32* overexpression lines *BBX32-OX #5* and *BBX32* artificial microRNA lines *BX32-AMI #*3 resulted in late flowering under SD conditions (Tripathi et al., 2016). Further studies are required to elucidate these underlying mechanisms.

## 4 Gibberellin-dependent regulation

In SD, rapid GA synthesis begins only when plants have been growing for a reproductive phase, and promotes flowering by facilitating floral meristem identity *LEAFY (LFY)* transcription via an independent *FT* pathway (Wilson et al., 1992; Eriksson et al., 2006). At the juvenile stage, plants inhibit flowering by suppressing the gibberellin response; at the adult phase, plants inhibit flowering by suppressing the gibberellin content; and at the critical stage of floral transition, flowering is promoted by rapidly increasing GA synthesis and response (Eriksson et al., 2006).

The exogenous application of GAs accelerates flowering in wild-type *A. thaliana*, particularly in SD (Langridge, 1957). Genetic analysis has suggested that GA has the most important function in flowering under SD conditions (Porri et al., 2012).

The *GA1* gene encodes the first enzyme involved in GA biosynthesis and regulates GA biosynthesis at an early stage; *ga1-3* did not flower in SD unless given exogenous GA, and weakly flowers late in LD (Wilson et al., 1992). *ga1-3* had a significantly stronger effect on flowering under SD than under LD, possibly because the photoperiodic pathway masked the effects of gibberellins in *ga1-3* under LD conditions (Reeves and Coupland, 2001). It has been shown that overexpression of *SOC1* or simultaneous inactivation of two GA-responsive GATA transcription factors, GATA NITRATE-INDUCIBLE CARBONMETABOLISM INVOLVED (GNC) and GNC-LIKE/CYTOKININRESPONSIVE GATA FACTOR1 (GNL) could rescue the flowering phenotype of the *ga1-3* plants in SD conditions (Moon et al., 2003; Richter et al., 2010). In contrast, SPINDLY (SPY) negatively regulates GA signaling, and *spy-1* is flowered early in SD (Jacobsen and Olszewski, 1993). The enzyme GIBBERELLIN 2 OXIDASE 7 (GA2ox7) catabolizes active GAs, and transgenic plants SUCROSE TRANSPORTER 2 (SUC2)*:GA2ox7* or *KNAT1:GA2ox7* were constructed to specifically express *GA2ox7* in vascular or shoot apical meristems. flowered later than the wild type, and *KNAT1:GA2ox7* flowered later, indicating that the role of GA in flowering under SD conditions is tissue specific, and that GA from shoot apical meristem tissues contributes more to flowering (Porri et al., 2012).

Three key GA signaling pathway components have been identified in *A. thaliana*: GA receptor GA INSENSITIVE DWARF1 (GID1), GA-response inhibitory protein factors DELLAs, and SLEEPY1 (SLY), of which GID1 and DELLAs have been reported to be associated with SD flowering; DELLAs inhibit all GA responses, whereas GID1 activates the GA response by binding and ubiquitinating DELLAs (Park et al., 2013).

DELLAs proteins play important roles as central regulatory nodes in the GA signaling pathway and are repressors that block GA signaling (Fleet and Sun, 2005). DELLAs proteins contain five members of the GRAS family of transcription factors: REPRESSOR OF GA1-3 (RGA), GA INSENSITIVE (GAI), RGA-LIKE 1 (RGL1), RGL2, and RGL3 (Sun and Gubler, 2004). *gaiΔ17*, *rgaΔ17*, *rgl1Δ17*, *rgl2Δ17*, and *rgl3Δ17* are GA-insensitive lines, collectively referred to as *dellaΔ17*. Different *dellaΔ17* proteins were identified by constructing *pSUC2:dellaΔ17*, *pFD:dellaΔ17*, and *pCLV3:dellaΔ17*, each of which has its own specific expression in the phloem, meristem, and shoot stem cell niches (Galvão et al., 2012). Under SD conditions, *dellaΔ17* expression in the meristem delayed flowering or did not flower, whereas *dellaΔ17* expression in the phloem and shoot stem had little effect on flowering time (Galvão et al., 2012). This differs from the finding that the expression of *rgl3Δ17* in the shoot stem does not affect flowering in LD (Galvão et al., 2012). It is hypothesized that DELLAs proteins regulate flowering tissues differently in LD and SD, and that they function mainly at the meristem in SD, which is consistent with the tissues in which GA2ox7 exerts its function (Porri et al., 2012; Galvão et al., 2012; Yu et al., 2012).

DELLA proteins and BOTRYTIS SUSCEPTIBLE1 INTERACTOR (BOI), BOI-RELATED GENE1 (BRG1), BRG2, and BRG3 (collectively referred to as BOIs) repress the GA response by interacting with and binding to the promoters of responsive GA genes (Park et al., 2013). The BOIs quadruple mutant *boiQ* and the DELLA pentuple mutant *dellaP* both flowered early in SD, consistent with their function in repressing the GA response (Park et al., 2013). The bHLH transcription factor MYC3 stabilized by DELLAs in SD inhibits flowering by inhibiting CO binding to *FT* and *myc3* early flowering in SD (Bao et al., 2019).

In addition, NO FLOWERING IN SD (NFL) is an obligate factor for the induction of flowering (Sharma et al., 2016) and promotes flowering by responding to gibberellins, which are active upstream of the GA signaling pathway; NFL belongs to the basic helix-loop-helix transcription factors, and its mutant, *nfl*, fails to flower in SD, but can flower by externally applying GA_4_ or by transferring *nfl* into the DELLA quadruple mutant *rga/gai/rgl1/rgl2*, and is therefore hypothesized to be a key transcription factor necessary for *A. thaliana* evolve into a parthenogenetic LD plant (Sharma et al., 2016).

HDC1 and HDA19 are directly responsible for HDAC and transcriptional repression of two flowering repressor genes in the gibberellin signaling pathway, GASA5 and GA2OX6, which together form a multi-subunit complex that regulates flowering; *hdc1* and *hda19* flowered early under SD conditions (Ning et al., 2019). The early flowering of *svp-41* is associated with an increase in *GA20ox2 mRNA*, and it is possible that GAs progressively induce the expression of SOC1 under SD conditions, which represses *SVP* transcription and promotes flowering (Andrés et al., 2014). Therefore, gibberellin plays an important role in promoting flowering under SD conditions, and the meristem is the main tissue in which it exerts its promoting function.

## 5 Prospects

**Figure 2:**
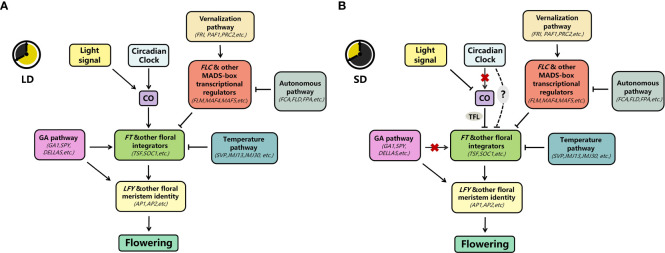
Key pathways for the photoperiodic regulation of flowering at 22°C **(A)** In LD, CO is mainly stabilized by light signals (PHYA, CRY1, etc.; see **Figure 1** for details) and circadian clock signals to promote *FT* transcription and thus flowering under LD; the gibberellin pathway promotes flowering either through *FT* or directly through *LFY*; the vernalization and autonomous pathways mainly inhibit flowering by inhibiting *FT* transcription through FLC and other MADS-box transcriptional regulators; and the temperature pathway inhibits *FT* transcription/sensitivity at 22°C by SVP and others. **(B)** In SD, CO is mainly degraded by light signals (PHYB, see **Figure 1** for details). The circadian clock does not promote *CO* transcription but represses *FT* transcription; the gibberellin pathway also does not promote *FT* transcription but directly promotes *LFY* transcription; the temperature pathway, vernalization pathway, and the autonomous pathway maintain a similar repression of *FT* transcription as LD under these conditions (→, promote; ⟞, inhabit; ‐‐‐, unclear; 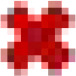, inactive).

In this paper, we reviewed the mechanisms underlying delayed flowering under SD conditions (**Figures 2A, B**). First, in contrast to its function in promoting flowering in LD, CO inhibits flowering dependent on FT in SD, at least through the TFL. Second, *A. thaliana* inhibits flowering by repressing *FT* transcription via multiple pathways. Finally, plants inhibit flowering by suppressing the response to or synthesis of gibberellins at different times before floral transition. Nevertheless, our understanding of the molecular mechanisms underlying SD-mediated delay in flowering in *A. thaliana* remains incomplete. Therefore, further in-depth investigation should be conducted.

### 5.1 Pathways and mechanisms involving CO

Typically, CO proteins in *A. thaliana* repress flowering in SD, as evidenced by the varying degrees of early flowering observed in several co-mutants (An et al., 2004; Datta et al., 2006; Redei, 1962; Balasubramanian et al., 2006; Fernandez et al., 2016; Luccioni et al., 2019; Ning et al., 2019; Yu et al., 2024; Zhang et al., 2015). The capacity of CO to bind to the *FT* promoter under SD conditions is constrained by competing factors (e.g., MYC3 and potentially others), and the activities of CO proteins that cannot bind to the FT promoter may be closely associated with the mechanism through which CO inhibits flowering. Recent studies have demonstrated that CO inhibits flowering via the TFL-FT pathway. However, it is noteworthy that the flowering phenotype of *co-9/tfl* was earlier than *co-9* but later than *tfl*, indicating a competitive inhibition of flowering between CO and TFL. It has been postulated that additional factors may operate in a *co-background* to promote flowering. The early flowering observed in the PHYB mutants *hy1* and *hy3* depends on *CO* (Putterill et al., 1995). In addition, PHYB degrades CO proteins through ubiquitination. However, there is a lack of biochemical evidence confirming whether early flowering in PHYB under SD conditions is caused by CO accumulation. It is also noteworthy that mutants containing elevated levels of CO proteins, such as *35S:3HA-CO*, *cop1*, and *spa1-7*, also exhibit early flowering phenotypes under SD conditions (Song et al., 2015; Laubinger et al., 2006; Jang et al., 2008). This differs from the function of CO in suppressing flowering under natural conditions, and further studies on the related mechanisms are required.

### 5.2 Mechanisms involving the FT pathway

The study of *FT* sensitivity is crucial for the inhibition of flowering by SD and the establishment of a precise quantification technique for plant sensitivity to FT is of paramount importance, cause *FT* levels cannot explain the similarity in flowering between LD and SD in some accessions (Kinmonth-Schultz et al., 2021). Studies have demonstrated that alterations in *FT* transcription within a specific range under SD conditions have a limited effect on flowering. Conversely, inducing *FT* expression during a period of heightened sensitivity to FT (ZT12-ZT20) is more likely to promote flowering in *A. thaliana* (Krzymuski et al., 2015). The mechanism underlying this period of heightened sensitivity depends on the circadian clock and remains to be elucidated. The sensitivity of the FT promoter can be regulated by its distance to its key elements, but the relevant trans-acting factor(s) and associated cis-element(s) remain unknown (Bao et al., 2019).

#### 5.2.1 Role of circadian clock

Several mutants that affect key components of the circadian clock have been identified, including *lhy-7, cca1-1, elf4, toc1-2, lwd1/lwd2*, and *cdf1/2/3/5* (Lin et al., 2019; Niwa et al., 2007; Park et al., 2016). Fornara et al. (2009) observed early flowering phenotypes and upregulation of *FT* transcription, indicating that the circadian clock pathway generally inhibits flowering under SD conditions by repressing *FT* transcription (**Figure 1**). Nevertheless, the precise mechanisms by which other components inhibit *FT* transcription and flowering remain unclear, except for LHY, which has been the subject of extensive research (Park et al., 2016).

#### 5.2.2 Role of red light

Several mutant studies have indicated that red light plays a role in the regulation of SD light suppression during flowering. Early flowering of the red-light receptor PHYB mutant phyB-5 is not associated with FT transcription and is independent of the GA pathway (Blázquez and Weigel, 1999). However, the early flowering phenotype of *srr1* and *hrb1* mutants of the red-light pathway has been observed to suppress flowering by repressing *FT* transcription (Johansson and Staiger, 2014; Kang et al., 2007). This suggests that the mechanism by which red light inhibits flowering is complex and requires further investigation.

#### 5.2.3 Mechanisms of FT inhibition of plant response to GA

Under SD conditions, GA promotes flowering independently of the *FT* pathway by binding to the GA response element in the *LFY* promoter and promoting its transcription (Blázquez et al., 1998; Eriksson et al., 2006). However, when GA_4_ was externally applied under SD conditions, *ft-1* flowered earlier than Col (Wang et al., 2009) and the *ft/tsf* double mutant flowered later than Col (Porri et al., 2012), suggesting that deletion of *FT* increased GA sensitivity of the plants, whereas deletion of both *FT* and *TSF* decreased GA sensitivity of the plants; however, the mechanism remains to be investigated.

### 5.3 Mechanisms involved in the GA pathway

#### 5.3.1 Unstudied key components of the GA pathway with flowering

The GA receptor GID1 has three homologous genes in *A. thaliana*: GID1a, GID1b, and GID1c, which are functionally redundant in regulating the GA signaling pathway (Griffiths et al., 2006). The *gida-1/gidb-1/gidc-1* triple mutant does not exhibit flowering under LD conditions, continuous light, or gibberellic acid (GA_3_) (Griffiths et al., 2006). Although there is a paucity of data regarding the flowering of this mutant under SD conditions, it probably does not flower. Further studies are needed to verify this hypothesis. Moreover, the flowering function of SLY1, a pivotal component of the GA pathway, under SD conditions, remains to be elucidated.

#### 5.3.2 Mechanism of co-regulation of flowering by GA and sucrose

Although GA_4_ plays a significant role in the flowering process, there was not a simple linear relationship between GA4 and *LFY* transcripts during the vegetative phase. Until the transition to the reproductive phase, when GA4 synthesis commences in substantial quantities and *LFY* transcripts increase, which may be associated with the varying levels of sucrose at different times (Eriksson et al., 2006), the mechanism by which GA and sucrose interact to stimulate flowering in SD remains uninvestigated.

### 5.4 Suggestions for crop breeding

Flowering is an important trait for improving crop yields (Carrera et al., 2024). *A. thaliana*, as a model plant, still has the ability to mine potential reference gene resources. In this article, it is summarized that *A. thaliana* achieves the suppression of *FT* under SD through multiple pathways, and the suppression of GA by the plant at the juvenile stage. So in the future, we can mine breeding resources from these two aspects in SD. Firstly, we can explore the genes that are the main regulators of FT in SD, such as mutants with extreme early flowering phenotypes, such as *elf3*, *elf8*,*cry1-L407F*(gain of function), etc, and mutants with flowering phenotypes only in SD, such as *vil1*, *vip4-2*, *prr7-11*, *vip5-1*, etc. Secondly, the research on the juvenile stage regulation of gibberellin in *A. thaliana* under SD condition is in-depth,exploring more primary repressors in this period is the most important task. Modulating them to access different seed resources through biotechnology (e.g., CRISPR-Cas) is more efficient.
